# Heredity of Color Blindness

**Published:** 1879-01

**Authors:** 


### HEREDITY OF COLOR-BLINDNESS.

The knowledge of the hereditary character of color-blindness is as old as that of the existence itself of color-blindness Indeed, the first two cases of Daltonism scientifically known. were two brothers.

Later, when a large number of cases of color-blindness were reported, and a greater amount of attention directed to the subject, observations in regard to cases of Daltonism perpetuated by inheritance, accumulated to such an extent that it became possible to establish some fixed law in regard to the mode of inheritance.

A law of inheritance, as for Daltonism, has, especially of late, been promulgated by Prof. Horner, of Zurich, according to which the color-blindness of the grandfather descends to the grandchildren.

According to Horner's law we find that color-blind men have color-seeing children, sons as well as daughters, and that the sons of these daughters are again color-blind,

However, the germ of inheritance is carried into families by the women, a fact the more striking, since they themselves possess a high degree of immunity from color-blindness. It is, therefore, possible for a man with a normal color faculty, in none of the members of whose family has there ever been a Daltonist, to have color-blind sons when he marries a daughter whose father was a Daltonist. From the knowledge of this fact we are enabled also now to better understand certain other observations. For example, the numerous cases of color-blindness occurring in the families of the mother of a Daltonist. The peculiarity of this law becomes still more interesting when we learn that it holds equally in regard to the inheritance of other physiological peculiarities. Thus we know that in some families the tendency of hæmorrhages is inherited according to this law, while again in other families, according to Pagenstecher, night-blindness extends in the same way. It would seem quite probable that this law may possess a general applicability to the theory of inheritance; at least from the above facts it would be of the greatest importance for physicians, especially family physicians, who have the opportunity of knowing and observing the families of their clients during different generations, to test more closely the scope of this important law,

Naturally this law does not exclude the possibility of other ways of transmitting Daltonism. Whether the relationship of parents can have an influence upon the development of color-blindness, as occurs in other defects, I do not attempt to decide.—*Prof. Magnus, Color-blindness, 1878.*

